# Upper eyelid contour measurement in an Asian population using Bézier curve analysis

**DOI:** 10.1371/journal.pone.0316714

**Published:** 2025-06-03

**Authors:** Da Young Shin, Won-Kyung Cho, Ji-Sun Paik, Suk-Woo Yang

**Affiliations:** 1 Department of Ophthalmology, Eunpyeong St. Mary’s Hospital, College of Medicine, The Catholic University of Korea, Seoul, Republic of Korea; 2 Department of Ophthalmology, Uijeongbu St. Mary’s Hospital, College of Medicine, The Catholic University of Korea, Seoul, Republic of Korea; 3 Department of Ophthalmology, Yeouido St. Mary’s Hospital, College of Medicine, The Catholic University of Korea, Seoul, Republic of Korea; 4 Department of Ophthalmology, Seoul St. Mary’s Hospital, College of Medicine, The Catholic University of Korea, Seoul, Republic of Korea; Wrocław University of Environmental and Life Sciences: Uniwersytet Przyrodniczy we Wroclawiu, POLAND

## Abstract

**Purpose:**

To define and quantify the upper eyelid contour of normal Asian adults using a software program utilizing Bézier curves.

**Methods:**

Eighty eyes of 80 healthy Korean subjects were included in this study. The Bézier curve tool of Image J software was used to extract the upper eyelid contours. The x and y coordinate values of the four points of the Bézier curve were analyzed using graphical analysis software in Matlab. Various parameters were measured automatically using a custom-written software algorithm.

**Results:**

The Bézier curves showed an excellent level of inter-rater reliability (intraclass correlation coefficient of 0.97 for absolute agreement, two-way random effects model, 95% CI 0.94 to 0.98). The Bézier curve for Asians was drawn from the medial palpebral commissure to the lateral canthus. The eye width was 24.8±1.8 mm in women and 25.3±2.0 mm in men. The contour peak was located at 1.7±1.0 mm temporally from the pupil center in women and 1.5±1.3 mm in men, and the height was 4.7±0.7 mm in women and 3.8±0.9 mm in men. The palpebral fissure obliquity (angle of the line connecting the medial canthal end to the lateral canthal end) was 9.98±3.07° in women and 7.52±2.89° in men.

**Conclusion:**

The upper eyelid contour was measured simply and efficiently using a Bézier curves function. The main features of Asian upper eyelid contour are that the inner part of the medial canthus is covered by the eyelids, and the lateral endpoint is located higher than the medial endpoint.

## Introduction

The objective assessment of eyelid contour is essential for diagnosing diseases or planning treatments in the oculoplastic field. The margin reflex distance (MRD1) is the most frequently used parameter and can be measured manually [1,2]. However, this measurement provides information only about the center of the eyelid [3]. In comparison, multiple mid-pupil-to-lid margin distances (MPLD) measured at 15-degree intervals from temporal to nasal offer a more comprehensive and reliable measurement for evaluating the entire eyelid contour [4,5].

The parameters for eyelid contour analysis are based on distance measurements. The simplest method is to use a portable ruler for direct measurements in the clinic room. However, this method allows measurement of only a few simple parameters such as MRD1, and the measured values can vary significantly depending on the measurer [6,7]. Therefore, such direct manual measurement exhibits low repeatability and reproducibility [6,8]. This limitation has been improved by using digital photos, with the use of digital calipers allowing for more delicate and diverse distance measurements [9–12]. Although previous studies utilized image analysis programs such as Image J and Adobe Photoshop [12–16] to employ digital tools for specific applications such as eyelid contour analysis, the procedures required multiple preprocessing steps and user manipulation, which can be time-consuming and laborious.

Recently, a novel approach to assessing eyelids using a Bézier curve was introduced [17,18]. A Bézier curve is a mathematically described curve used in computer graphics and related fields. They are used to model smooth curves that can be scaled indefinitely. Several researchers reported that the Bézier function can be very useful for assessing eyelid contour [17,19,20]. Nonetheless, Bézier curve analysis can still be unfamiliar and challenging for ophthalmologists or clinicians, possibly because of the mathematical processing of the Bézier function and the challenges in its application within image analysis software. Furthermore, there are few studies that have analyzed Asian eyelids using Bezier curves, and the eyelid shapes of Western and Asian people have different characteristics [21]. When performing such analysis, the reference line or methods may differ from those described in previous studies conducted on Western populations.

In the present study, we describe a method of utilizing Bézier curves for eyelid analysis in Asians, and introduce a new image analysis algorithm we have developed. We also assess the reliability of our new method.

## Methods

### Study subjects

This study included eighty eyes of 80 healthy patients (40 women and 40 men) who visited the University of Seoul St. Mary’s Hospital between 01/01/2022 and 31/07/2023. They visited the ophthalmology clinic for routine care of dry eye syndrome or tearing. Patients with systemic disease or previous surgery that could affect eyelid contour were excluded. This study was approved by the Institutional Review Board of the Catholic University of Korea, Seoul, Republic of Korea (Ethical approval no. XC24RIDI0039, 12/07/2024). The participants medical records were accessed from the University of Seoul St. Mary’s Hospital in 13/07/2024. The data of the patients were collected starting from 13/07/2024. Patients were asked to sit and fixate on a distant target. Photographs with flash were taken in the primary position by the same person (SW-Y) using a digital camera (Nikon D 90; Nikon Corp., Tokyo, Japan). All eligible participants were included in the chart review. All procedures adhered to the principles of the Declaration of Helsinki. As the data were retrospectively evaluated pseudonymously and were solely obtained for treatment purposes, the requirement for informed consent was waived by the Ethics Committee of Seoul St. Mary’s Hospital, Korea.

### Lid contour analysis

**Fig 1:**
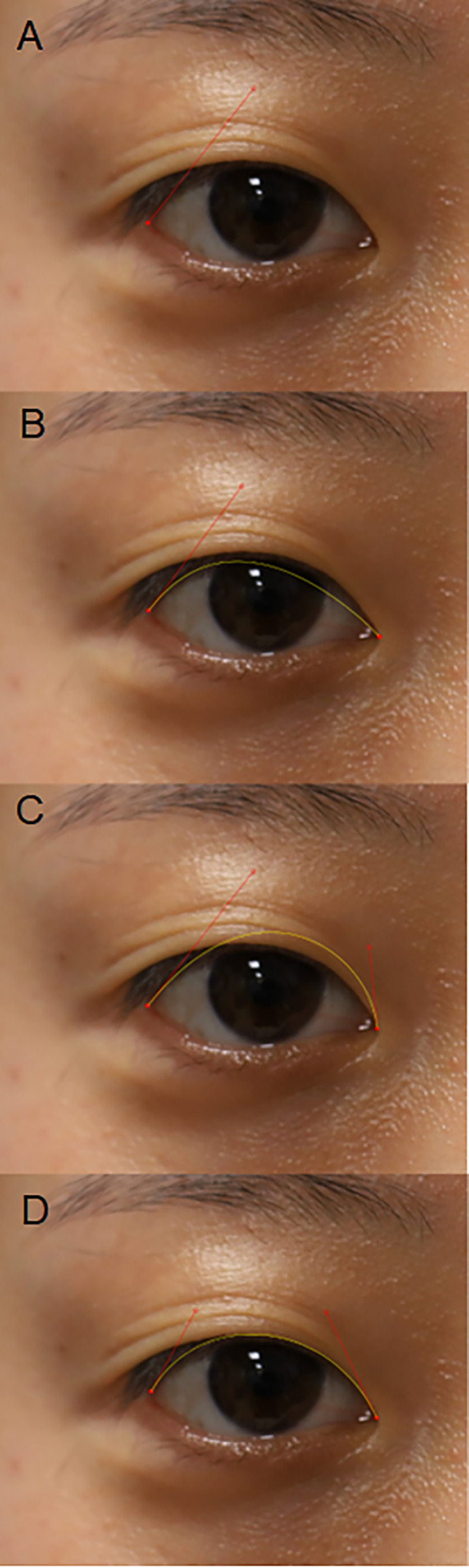
Bézier curve. **A.** Click the lateral end point (lateral canthus) and drag it somewhere between eyebrow and upper eyelid. Hollow red circle is first control point. **B.** Clicks the medial end point (medial palpebral commissure), which generates a yellow curve. **C.** Click on the medial end point and drag upward to create a second control point. **D.** The Bézier curve that best fits the eyelid margin.

**Fig 2:**
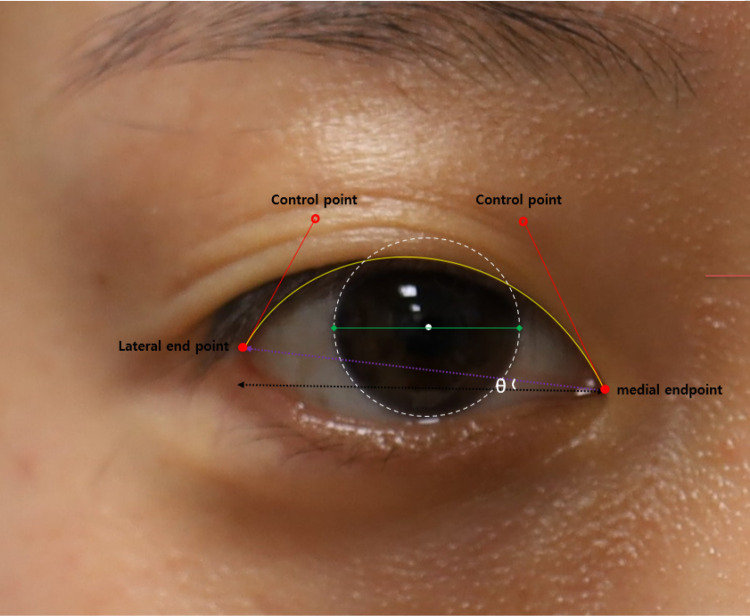
Parameters in Bézier curve. Two red dot are lateral and medial endpoint. lateral end point (lateral canthus) and drag it somewhere between eyebrow and upper eyelid. Hollow red circles are control points. White dot circle is cornea margin. White dot spot is pupil center. The angle(θ) formed by the line connecting the outer and inner end point (purple dot line) and the horizontal baseline (black dotted line) was defined as fissure obliquity(θ).

The upper eyelid contour was assessed using the National Institutes of Health image-analysis software (ImageJ version 1.40; available at http://rsb.info.nih.gov/ij/index.html; National Institutes of Health, Bethesda, MD). The Bézier plugin from the ImageJ software was employed to fit the best curve to the upper lid contour. The procedure for drawing a Bézier curve is as follows: when measuring the right eye, the examiner clicks the lateral endpoint (lateral canthus) and drags it somewhere between the eyebrow and upper eyelid to serve as the first control point; the location of this point can be modified later (Fig 1A). Next, the examiner clicks the medial endpoint (medial palpebral commissure), generating a yellow (pre-selected color) curve (Fig 1B). Subsequently, a click on the medial endpoint is made and it is dragged upward to establish a second control point. The examiner then adjusts the two control points to determine the curve that best fits the eyelid margin (Fig 1C). This resulting curve is a Bézier curve (Fig 1D). The technique has been previously described and applied in a prior investigation [17,18]. Numerical pixels corresponding to corneal diameter are converted to millimeters, assuming a white-to-white corneal diameter of 12.0 mm on the basis of previous reports [22]. The corneal diameter is represented by a green line, and the pupil center is manually marked with a white dot, as shown in Fig 2. The polar coordinate data of four reference points are extracted, with the pupil center point serving as the origin.

The collected data are imported into statistical software for graphical analysis (Matlab, MathWorks, Natick, Massachusetts, USA). Using the Bézier function and software algorithm (S1,S2,S3) developed by author D.Y.S., various index values of the eyelid can be automatically measured and calculated. Eye width is defined as the distance between the x-coordinates of both endpoints of the eyelid curve. MRD1 is equivalent to the MPLD at 90°. The contour peak was defined as the location when the y value of the bézier curve was largest. The palpebral fissure obliquity (**θ**°) is defined as the angle between the line connecting the two end points (medial and lateral canthal ends) and the horizontal line, which is also automatically calculated using a coordinate system (S1 Appendix).

**Fig 3:**
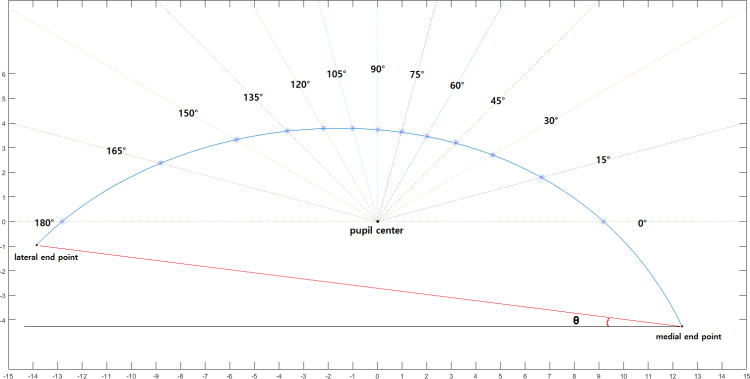
Digital image analysis. A Bézier curve is drawn in Matlab software program using the coordinate values obtained from image J. And radial lines 15 degrees apart from the pupil center are marked. The observer marked the intersections of the radial line on the lid margin edge, and the software automatically calculated the coordinates of the intersection with the pupil center as the origin. The angle(**θ**) formed by the line connecting the outer and inner end point (red dot line) and the horizontal baseline (black line) was defined as fissure obliquity.

In the case of MPLD, 13 first-order function equations corresponding to radial and vertical lines (180°,165°, 150°, 135°, 120°, 105°, 90°, 75°, 60°, 45°, 30°, 15°, 0°) are created (Fig 3). The radial MPLD is automatically calculated using a software algorithm that finds the intersection with the Bézier function. The intersection point is marked with a ‘✴’ in Fig 3 (S2 Appendix). The temporal-to-nasal mid-pupil lid distance ratios, which form the same angles from the midline, such as ratios of 105:75, 120:60, 135:45, 150:30, 165:15 and180:0 are also calculated. Two of the authors (D.Y.S., S.W.Y.) independently isolated the upper lid contours of 80 subjects and obtained the above-described measurements.

### Statistical analysis

Data are presented as mean and standard deviation. The independent *t*-test was used to compare two groups (male and female). A value of p < 0.05 was taken to indicate statistical significance. All statistical analyses were performed using SPSS for Windows (v. 24.0; IBM Corporation, Armonk, NY, USA). A Bland-Altman plot was used to test the inter-observer variability of the height of the contour peak determination. The degree of agreement between the two contours was also estimated by calculating the intraclass correlation coefficient.

## Results

The mean contour peak height was 4.29 mm (SD 0.92), and showed a good level of inter-rater reliability (intraclass correlation coefficient of 0.98 for absolute agreement, two-way random effects model, 95% CI 0.94 to 0.98). The Bland–Altman plot did not reveal a significant difference between measurements of counter peak height (Fig 3).

**Fig 4:**
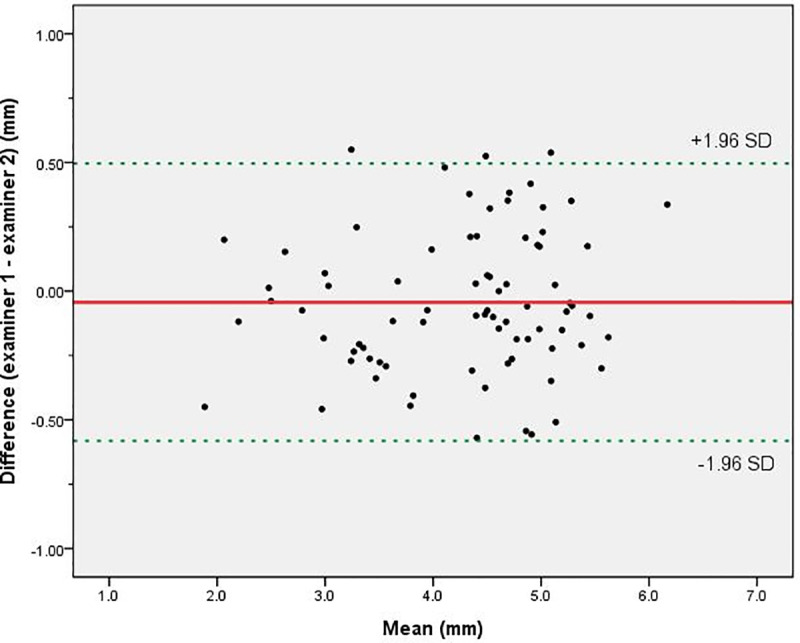
Bland-Altman plot of the differences of the contour peak height extracted by two independent examiners.

**Fig 5:**
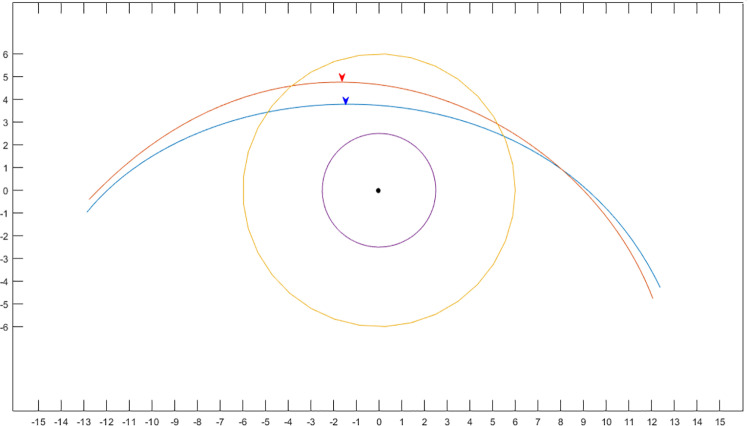
Upper eyelid contour drawn by Bézier function. That was extracted from our sample and plotted in polar coordinates. Blue curve represents male contour, and red curve represents the female contour. The yellow circle is cornea. The purple circle is pupil margin. The black dot is the pupil center. The arrowhead indicates the location of contour peak.

The demographics features of the patients are shown in Table 1. The mean age was 28.80±5.59 years in women and 28.18±6.06 years in men. The mean eye width was 24.82±1.82 mm in women and 25.25±2.00 years in male. The MRD1 was 4.62±0.70 mm in women and 3.77±0.93 mm in men (p < 0.001). The mean contour peak height was 4.74±0.68 mm in women and 3.84±0.93 in men (p < 0.001). The contour peak point was located 1.67±1.02 mm temporally away from the pupil center in women and 1.49±1.33 mm away in men (p = 0.501, Fig 4). The palpebral fissure obliquity was significantly larger in women (9.98±3.07°) than in men (7.52±2.89°, p < 0.001). Table 2 shows radial MPLD from 15° to 165° for both men and women. The temporal-nasal mid-pupil eyelid distance ratios were higher in women than in men, but there was no statistically significant difference in the total ratios (p = 0.106, Table 3). Fig 5 demonstrated the pupil margin and eyelid contours of males and females in a single image (S3 Appendix).

**Table 1:** Summary of results in upper lid contours in Asian subjects.

	Total	Female	Male	p value
**Age (year)**	28.49±5.80	28.80±5.59	28.18±6.06	0.633
**Eye width (mm)**	25.03±1.90	24.82±1.82	25.25±2.00	0.314
**Palpebral Fissure Obliquity(°)**	8.75±3.19	9.98±3.07	7.52±2.89	**<0.001**
**MRD1 (mm)**	4.19±0.92	4.62±0.70	3.77±0.93	**<0.001**
**Contour peak location (horizontal)**	1.58±1.17	1.67±1.02	1.49±1.33	0.501
**Contour peak height (mm)**	4.29±0.92	4.74±0.68	3.84 ± 0.93	**<0.001**

**Table 2:** Midpupil lid distances of the right eye from 0 to 180 degrees.

	MPLD	MPLD	MPLD	MPLD	MPLD	MPLD	MPLD	MPLD	MPLD	MPLD	MPLD	MPLD	MPLD
	**180°**	**165°**	**150°**	**135°**	**120°**	**105°**	**90°**	**75°**	**60°**	**45°**	**30°**	**15°**	**0°**
Female	12.15 ± 0.83	9.73 ± 0.90	7.66±0.86	6.28 ± 0.81	5.40±0.75	4.87±0.72	4.62±0.70	4.59±0.72	4.78±0.75	5.23±0.81	6.00±0.91	7.24±1.08	9.12±1.21
Male	11.97±1.02	8.89±1.29	6.55±1.29	5.18±1.16	4.38±1.04	3.95±0.97	3.77±0.93	3.79±0.94	4.03±0.99	4.53±1.07	5.44±1.22	6.94±1.39	9.23±1.57
Total	12.04±0.93	9.31±1.17	7.11±1.21	5.73±1.13	4.89±1.03	4.41±0.96	4.19±0.92	4.19±0.92	4.41±0.94	4.88±1.00	5.72±1.10	7.09±1.24	9.18±1.39

**Table 3:** Temporal-to-Nasal Midpupil Lid Distance Ratios.

Gender	MPLD 180/0	MPLD 165/15	MPLD 150/30	MPLD 135/45	MPLD 120/60	MPLD 105/75	Ratio total
Total	1.33±0.23	1.34±0.25	1.26±0.19	1.18±0.14	1.11±0.08	1.05±0.04	1.35±0.14
Female	1.31±0.17	1.37±0.22	1.30±0.18	1.21±0.13	1.13±0.08	1.06±0.04	1.36±0.13
Male	1.34±0.27	1.32±0.27	1.23±0.20	1.15±0.14	1.09±0.08	1.04±0.04	1.34±0.14
p value	0.680	0.341	0.107	**0.048**	**0.023**	**0.021**	0.106

## Discussion

In this study, we used the Bézier function to extract the curve of the eyelid and developed an algorithm in MATLAB to automatically calculate various distances of the eyelid contour. We analyzed the eyelids of normal adult Asians, specifically Koreans. When plotting the Bézier curve, the most important difference between our study and previous studies conducted on Western subjects was in the location of the inner endpoint of the eyelid curve. The medial endpoint of the eyelid arch in Asians is set at the medial palpebral commissure, whereas in Western populations it is designated at the upper lacrimal punctum. The eyelids of Asians are anatomically and structurally different from those of Westerners [17,19,20]; the canthus of a Caucasian eye is exposed completely, whereas the inner part of the medial canthus of the Asian eye is veiled by the epicanthus [23,24]. The epicanthus or epicanthal fold is a skin fold that descends along the side of the nose from the upper eyelid to the medial aspect of the lower eyelid. It covers the innermost edge of the eyelid, allowing the eyelid contour to smoothly extend to the curved inner edge. Another important difference is the upward tilt of the eyelids. The palpebral slant is the direction of the slant of a line that goes from the outer corner of the eye to the inner corner. In our study, the palpebral slant was quantified by palpebral fissure obliquity, which was more prominent in females than in men. Previous studies comparing palpebral fissure obliquity among different races reported values of 4.6° for Caucasians, 9.4° for Japanese, and 9.6° for Indians [24]. A study on Korean subjects reported values of 7.1° for women and 6.0° for men [5]. In comparison, our study showed a large inclination angle (9.98° for women, 7.52° for men) and showed more differences between genders (p < 0.001). This is probably because our study included only relatively young people (20–39), in contrast to previous study that included people aged 20–80. In addition to palpebral fissure obliquity, we described other parameters such as width, MPLD, contour peak, and temporal-to-nasal MPLD ratio.

The parameters for eyelid contour analysis are based on distance measurements. These distances can be measured manually, but such measurements can be imprecise and can be highly variable. There are also limitations to the number of parameters that can be realistically measured, and the method is not appropriate for evaluating the entire eyelid [3]. Digital photographs allow more parameters to be measured more accurately using digital calipers.

Image analysis programs used for this process include Image J and Adobe Photoshop [12–16]. These tools are already widely used in a variety of fields and are highly accessible, but their application in the field of ophthalmology requires multiple processes. There have been attempts to develop automatic image processing technology to extract eyelid contour curves, but they were not entirely successful, coming across various difficulties [12,25]. Milbratz introduced a method to automatically calculate MPLD using MATLAB-based graphical analysis software and an x, y coordinate system [4]. MATLAB is a program that automatically calculates numerical values, and many researchers have used it to analyze eyelid contour [5,26,27]. However, it was still necessary to directly mark the relevant point on the eyelid. Other researchers developed their own in-house digital image processing software designed to facilitate assessment of the eyelid contour [28]. Nevertheless, the use of self-developed programs may present challenges, as they may not be readily accessible or available for free use by other groups.

In this study, we used Bézier curves to simplify manual work such as drawing eyelid outlines directly with a mouse or marking relevant points on the digital photo. This method provided improved accuracy and reduced variability in comparison with manual measurements. Bézier curves were developed in 1962 by Pierre Bézier to represent curves in the field of automotive design [29,30]. Subsequently, their application expanded into the field of computer science, where they have been widely used in graphic design, computer-aided design, animation, 3D modeling, and various other areas [31,32]. Bézier curves have now become an important mathematical tool in computer graphics and design, and since the 2000s, the development of computer technology and medical image processing has led to the application of Bézier curves in the medical field [33]. In the field of ophthalmology, Goldbert and Garcia introduced Bézier curves for assessing eyelid contours [17,18] and Huelin developed metrics to assess the symmetry and contour peak location of eyelid contours using Bézier curves [19]. Although Sales-Sanz and Liu used Bézier curves to evaluate post-eyelid surgery outcomes [21,34], they are not yet widely employed in the field of oculoplastic surgery.

The process of drawing Bézier curves along the eyelid takes just a few seconds. Two control points are sufficient for drawing a Bézier curve along the eyelid line. Our Bézier curve-based software can be easily and simply used, even for beginners, without highly specific equipment.

After drawing a Bézier curve in ImageJ and obtaining only four points, all subsequent numerical analyses of the eyelid are automatically calculated using MATLAB software. Additionally, the Bézier function allows eyelid contour to accurately replicate again with minimal data. We showed this work in Fig 5 and S3 Appendix. In this study, we describe only a few representative parameters used to evaluate the eyelid; however, once the algorithm is developed, it enables easy automatic calculations. Clinical ophthalmologists could potentially find it difficult to use the mathematical image processing and algorithms, but we will provide the algorithms as open source online software in the MATLAB programming environment (S1 Appendix). Even without understanding the mathematical processing of curves, the algorithm can be easily downloaded and used. Moreover, this algorithm can be freely added to, modified, and expanded by others. In this study, we did not use new specialized software, instead using commonly available tools in the medical field, making it easy to replicate and apply our methods. Image J and MATLAB are imaging processing programs that are extensively used in the medical field. We introduced a method for obtaining measurements that used the two programs together and created the algorithm applied here that facilitates a more straightforward evaluation of the eyelid contour.

There are some limitations to our study. First, the study only included Korean subjects. Second, since the eyelid assessment method requires digital imaging processing, it may not be easily applicable in clinical practice. Drawing the Bézier curve and marking the pupil center are performed manually, and there may therefore be a learning curve associated with the technique and the examiner’s experience may be important in obtaining the measurements. Image processing techniques that automatically determine the curve or pupil center point may solve this issue in the future. Lastly, the lengths measured in our study were not the actual lengths because the horizontal corneal diameter was set to 12 mm and converted to a length ratio. However, because evaluation of eyelid deformity uses a distance ratio rather than the actual length, this method may be more appropriate for diagnosing eyelid disorders.

## Supporting information

S1 Appendix MATLAB algorithm. (DOCX)

S2 Appendix MATLAB algorithm. (DOCX)

S3 Appendix MATLAB algorithm. (DOCX)

S4 Appendix Data. (XLSX)
