# Supplementary material for: Upper eyelid contour measurement in an Asian population using Bézier curve analysis
Source: PLoS One. 2025 Jun 3;20(6):e0316714. doi: 10.1371/journal.pone.0316714 (PMC12132956; doi:10.1371/journal.pone.0316714)
Supplement: S1 Appendix — (DOCX) [file pone.0316714.s001.docx]

Eye lid contour peak, eye width, Palpebral Fissure Obliquity (Bezier Curve)

Plot the Third-order Bezier curve specified by the control points p0 = [-13.15 -0.24], p1 = [-5.21 7.88], p2 = [8.53 5.51], p3 = [3 0], p4 = [12.48 -4.98]. Create a matrix with each row representing a control point.

| P=[ | -12.643 | -0.536 | ; | -9.964 | 6.214 | ; | 8.893 |
| --- | --- | --- | --- | --- | --- | --- | --- |
| 5.679 | ; | 12.321 | -5.464 | ]; |  |  |  |

Compute the Third-order Bernstein matrix B.

syms t

B = bernsteinMatrix(3,t);

Construct the Bezier curve.

bezierCurve = simplify(B*P);

Plot the curve adding the control points to the plot.

fplot(bezierCurve(1), bezierCurve(2), [0 1]) hold on

scatter(P(:,1), P(:,2),'filled') title('Third-order Bezier curve') hold off


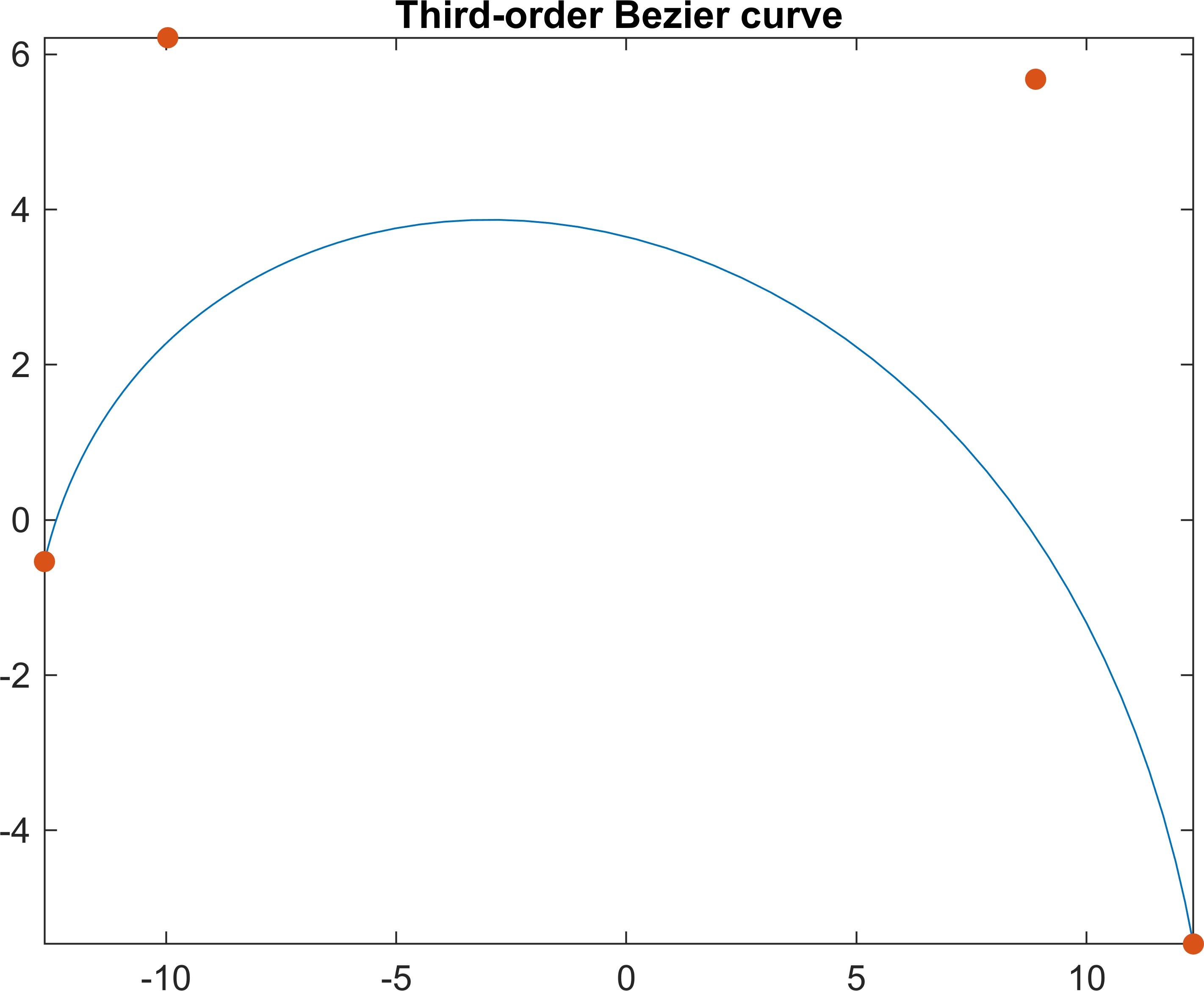


fig = gcf

fig =

Figure (2) with properties:

Number: 2 Name: ''

Color: [1 1 1]

Position: [680 458 560 420] Units: 'pixels'

Show all properties

h=gcf

h =

Figure (2) with properties:

Number: 2 Name: ''

Color: [1 1 1]

Position: [680 458 560 420] Units: 'pixels'

Show all properties

fplot(bezierCurve(1), bezierCurve(2), [0 1])


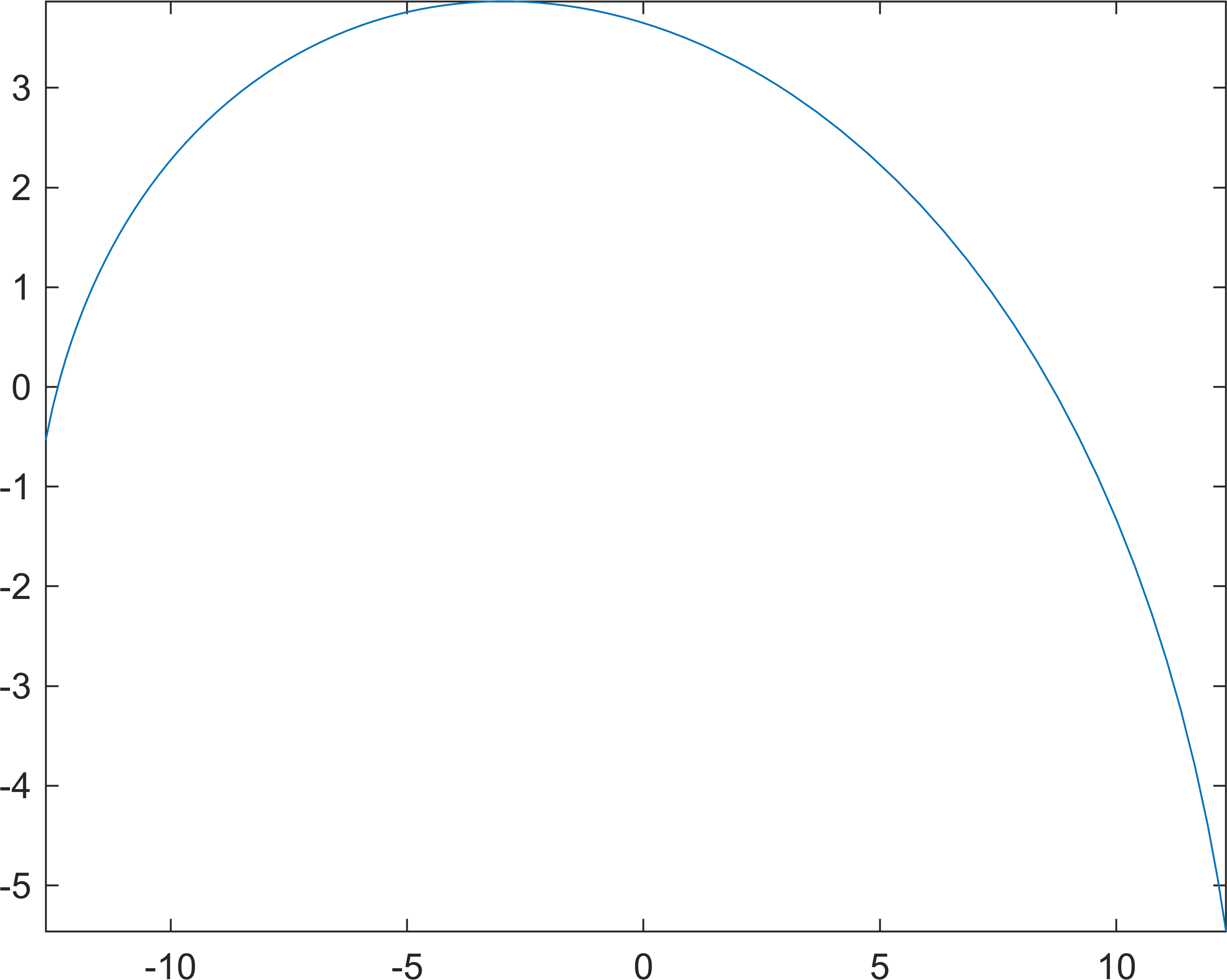


h = get(gca, 'children'); x = get(h(1), 'xdata');

y = get(h(1), 'ydata'); [y1 I1] = max (y)

y1 = 3.8668

I1 = 31

A=[x;y]

A = 2×66

| -12.6430 -12.5739 | -12.4984 | -12.4166 | -12.3286 | -12.2170 | -12.0970 | -11.9689 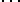 |
| --- | --- | --- | --- | --- | --- | --- |
| -0.5360 -0.3716 | -0.2101 | -0.0515 | 0.1041 | 0.2837 | 0.4592 | 0.6306 |
| x1=A(1,I1) |  |  |  |  |  |  |
| x1 = -2.7722 |  |  |  |  |  |  |

z=abs(x)

z = 1×66


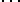
12.6430 12.5739 12.4984 12.4166 12.3286 12.2170 12.0970 11.9689

[X I2] =min (z)

X = 0.4419

I2 = 33

x2=A(1,I2)

x2 = -0.4419

y2=A(2,I2)

y2 = 3.7099

J=[y2,x1,y1]

J = 1×3

3.7099 -2.7722 3.8668

C =[x1,y1]

C = 1×2

-2.7722 3.8668

C is counter peak location.

w = P(4, 1) - P(1, 1);

l = P(1, 2) - P(4, 2);

angle_degrees = 90 - rad2deg(atan(w/l)); G = [x1,y1, w,angle_degrees]

G = 1×4

-2.7722 3.8668 24.9640 11.1669

G is [counter peak x coordinate, counter peak y coordinate , eye width, Palpebral Fissure Obliquity]

*nc.*
