# Supplementary material for: Upper eyelid contour measurement in an Asian population using Bézier curve analysis
Source: PLoS One. 2025 Jun 3;20(6):e0316714. doi: 10.1371/journal.pone.0316714 (PMC12132956; doi:10.1371/journal.pone.0316714)
Supplement: S2 Appendix — (DOCX) [file pone.0316714.s002.docx]

MLDP measurements

Plot the Third-order Bezier curve specified by the control points p0 = [-13.15 -0.24], p1 = [-5.21 7.88], p2 = [8.53 5.51], p3 = [3 0], p4 = [12.48 -4.98]. Create a matrix with each row representing a control point.

| P=[ | -12.828 | -0.662 | ; | -7.283 | 8.359 | ; | 9.186 |
| --- | --- | --- | --- | --- | --- | --- | --- |
| 5.545 | ; | 13.324 | -3.807 | ]; |  |  |  |

Compute the Third-order Bernstein matrix B.

syms t

B = bernsteinMatrix(3,t);

Construct the Bezier curve.

bezierCurve = simplify(B*P);

Plot the curve adding the control points to the plot. Z is MPLD 15' 30' 45' 60' 75' 90' 105' 120' 135' 150' 165'.

fplot(bezierCurve(1), bezierCurve(2), [0 1]) title('Third-order Bezier curve')


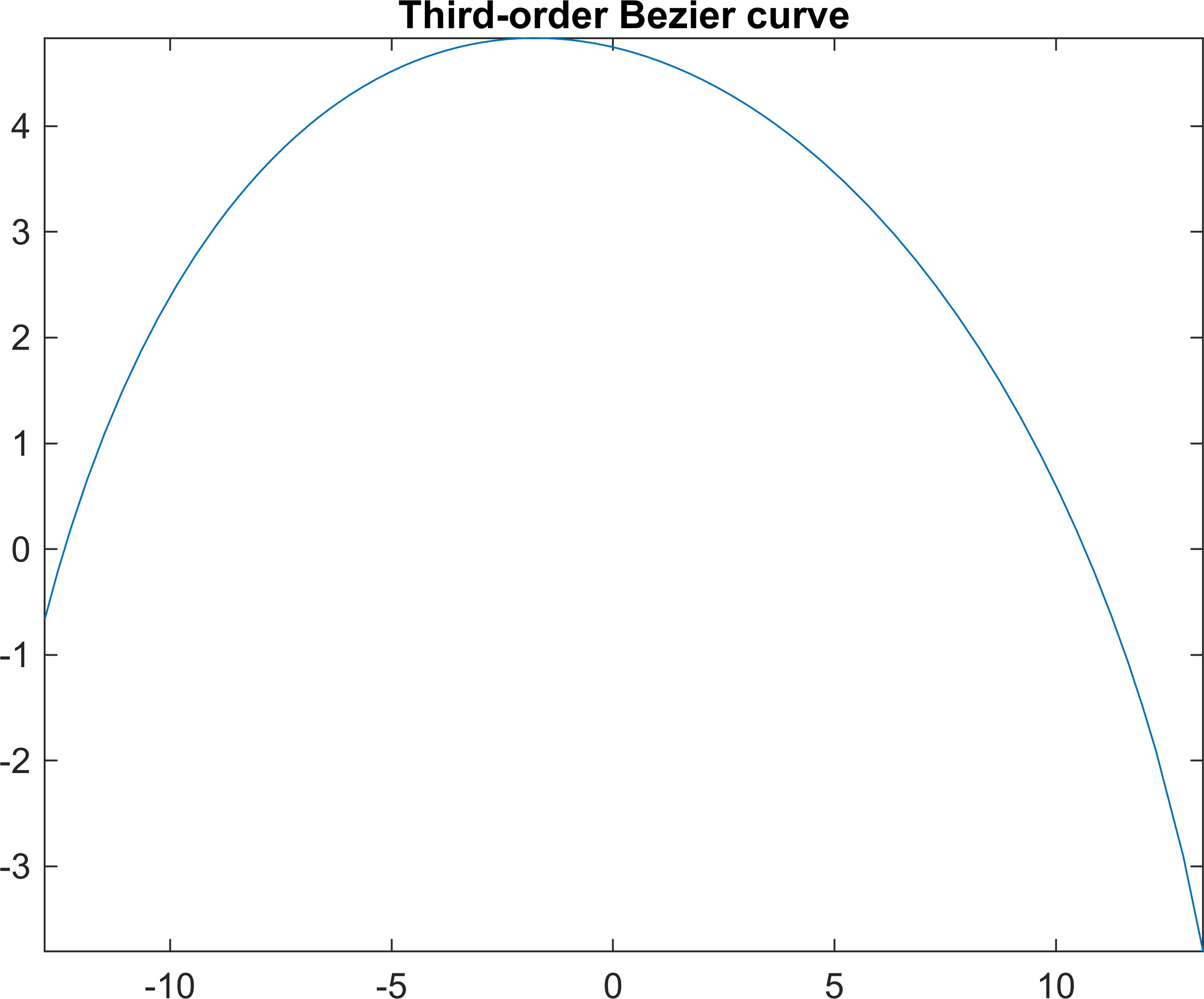


fplot(bezierCurve(1), bezierCurve(2), [0 1]) fig = gcf

fig =

Figure (1) with properties:

Number: 1 Name: ''

Color: [1 1 1]

Position: [680 458 560 420] Units: 'pixels'

Show all properties

h=gcf

h =

Figure (1) with properties:

Number: 1 Name: ''

Color: [1 1 1]

Position: [680 458 560 420] Units: 'pixels'

Show all properties

h = get(gca, 'children'); x = get(h(1), 'xdata');

y1 = get(h(1), 'ydata');

set(gca,'XTick',[-15:1:15])

set(gca,'YTick',[-10:1:10]) daspect([1 1 1])

xlim([-15 15])

ylim([-15 15]) hold on

y2=-0.2679*x

y2 = 1×59


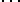
3.4366 3.3613 3.3219 3.2814 3.2322 3.1815 3.1293 3.0758

y3=-0.5774*x

y3 = 1×59


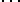
7.4069 7.2445 7.1596 7.0723 6.9662 6.8570 6.7446 6.6292

y4=-x

y4 = 1×59


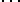
12.8280 12.5467 12.3997 12.2484 12.0648 11.8756 11.6810 11.4811

y5=-1.7321*x

y5 = 1×59


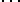
22.2194 21.7322 21.4775 21.2155 20.8975 20.5698 20.2327 19.8863

y6=-3.7321*x

y6 = 1×59


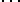
47.8754 46.8256 46.2768 45.7124 45.0271 44.3211 43.5947 42.8485

y7=3.7321*x

y7 = 1×59


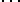
-47.8754 -46.8256 -46.2768 -45.7124 -45.0271 -44.3211 -43.5947 -42.8485

y8=1.7321*x

y8 = 1×59


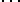
-22.2194 -21.7322 -21.4775 -21.2155 -20.8975 -20.5698 -20.2327 -19.8863

y9=x

y9 = 1×59


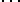
-12.8280 -12.5467 -12.3997 -12.2484 -12.0648 -11.8756 -11.6810 -11.4811

y10=0.5774*x

y10 = 1×59


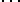
-7.4069 -7.2445 -7.1596 -7.0723 -6.9662 -6.8570 -6.7446 -6.6292

y11=0.2679*x

y11 = 1×59


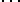
-3.4366 -3.3613 -3.3219 -3.2814 -3.2322 -3.1815 -3.1293 -3.0758

plot(x,y2,':',x,y3,':',x,y4,':',x,y5,':',x,y6,':',x,y7,':',x,y8,':',x,y9,':',x,y10,'

:',x,y11,':')

yline(0,':')

xline(0,':')

xlim([-15 15])

ylim([-5 7]) hold off

[xi,yi] = getpts


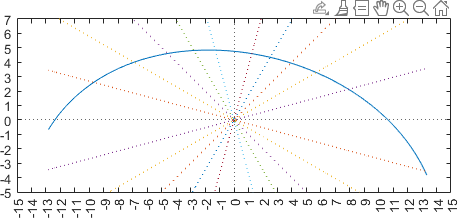


xi = 13×1

-12.3387

-9.6429

-7.0161

-4.6659

-2.7995

-1.3479

-0.0346

1.2788

2.5230


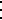
3.9055

yi = 13×1

-0.0369

2.6590

3.9724

4.5945

4.8018

4.8710

4.8018

4.5945

4.3180


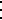
4.0415

A=[xi,yi]

A = 13×2

| -12.3387 | -0.0369 |
| --- | --- |
| -9.6429 | 2.6590 |
| -7.0161 | 3.9724 |
| -4.6659 | 4.5945 |

| -2.7995 | 4.8018 |
| --- | --- |
| -1.3479 | 4.8710 |
| -0.0346 | 4.8018 |
| 1.2788 | 4.5945 |
| 2.5230 | 4.3180 |
| 3.9055 | 4.0415 |


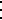


C=sqrt((xi).^2+(yi).^2)

C = 13×1

12.3388

10.0027

8.0626

6.5483

5.5583

5.0540

4.8020

4.7691

5.0011


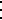
5.6202

Z=C'

Z = 1×13


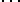
12.3388 10.0027 8.0626 6.5483 5.5583 5.0540 4.8020 4.7691

Z is measured MPLD.
