# Supplementary material for: Upper eyelid contour measurement in an Asian population using Bézier curve analysis
Source: PLoS One. 2025 Jun 3;20(6):e0316714. doi: 10.1371/journal.pone.0316714 (PMC12132956; doi:10.1371/journal.pone.0316714)

2-D Bezier Curve Male vs Female

Plot the Third-order Bezier curve specified by the control points p0 = [-13.15 -0.24], p1 = [-5.21 7.88], p2 = [8.53 5.51], p3 = [3 0], p4 = [12.48 -4.98]. Create a matrix with each row representing a control point.

| P1=[-12.869 | -0.972 |  | ; | -7.529 | 5.598 | ; | 7.375 | 6.139 | ; |
| --- | --- | --- | --- | --- | --- | --- | --- | --- | --- |
| 12.382 | -4.274 | ]; |  |  |  |  |  |  |  |

Compute the Third-order Bernstein matrix B.

syms t

B = bernsteinMatrix(3,t);

Construct the Bezier curve.

bezierCurve = simplify(B*P1);

Plot the curve adding the control points to the plot.

fplot(bezierCurve(1), bezierCurve(2), [0 1]) title('Eyelid curve')

hold on

Plot the fourth-order Bezier curve specified by the control points p2 = [-13.15 -0.24], p1 = [-5.21 7.88], p2 = [8.53 5.51], p3 = [3 0], p4 = [12.48 -4.98]. Create a matrix with each row representing a control point.

| P2=[-12.770 | -0.405 |  | ; | -3.345 | 9.527 | ; | 8.919 | 3.497 | ; |
| --- | --- | --- | --- | --- | --- | --- | --- | --- | --- |
| 12.061 | -4.764 | ]; |  |  |  |  |  |  |  |

Compute the Third-order Bernstein matrix B.

syms t

B = bernsteinMatrix(3,t);

Construct the Bezier curve.

bezierCurve = simplify(B*P2);

Plot the curve adding the control points to the plot.

fplot(bezierCurve(1), bezierCurve(2), [0 1]) hold on

Combine two curve

fig = gcf

fig =

Figure (3) with properties:

Number: 3 Name: ''

Color: [1 1 1]

Position: [680 458 560 420] Units: 'pixels'

Show all properties

h=gcf

h =

Figure (3) with properties:

Number: 3 Name: ''

Color: [1 1 1]

Position: [680 458 560 420] Units: 'pixels'

Show all properties

h = get(gca, 'children'); x = get(h(1), 'xdata');

y1 = get(h(1), 'ydata');

set(gca,'XTick',[-15:1:15])

set(gca,'YTick',[-6:1:6]) daspect([1 1 1])

xlim([-16 16])

ylim([-8 8]) hold on

Plot the circle 6mm radian circle cornea

center=[0 0];

r=6; N=100;

theta=linspace(0,6*pi,N); x=r*cos(theta)+center(1); y=r*sin(theta)+center(2); plot(x,y);

axis equal; hold on

%%%%%%%% Center of the circle

%%%%%%%%

%%%%%%%%

Radius of the circle

Number of dividing

%%%%%%%%

%%%%%%%%

%%%%%%%%

Angle of the circle(Radian)

x coordinate y coordinate

%%%%%%%%

%%%%%%%%

Plot the circle

Set aspect ratio equal

Combine two circle inner circle pupil.

center=[0 0];

r=2.5; N=100;

theta=linspace(0,2.5*pi,N);

%%%%%%%% Center of the circle

%%%%%%%%

Radius of the circle

%%%%%%%%

Number of dividing

%%%%%%%%

Angle of the circle(Radian)

x=r*cos(theta)+center(1);

y=r*sin(theta)+center(2); plot(x,y);

axis equal;

%%%%%%%% x coordinate

%%%%%%%%

y coordinate

%%%%%%%%

%%%%%%%%

Plot the circle

Set aspect ratio equal

hold on


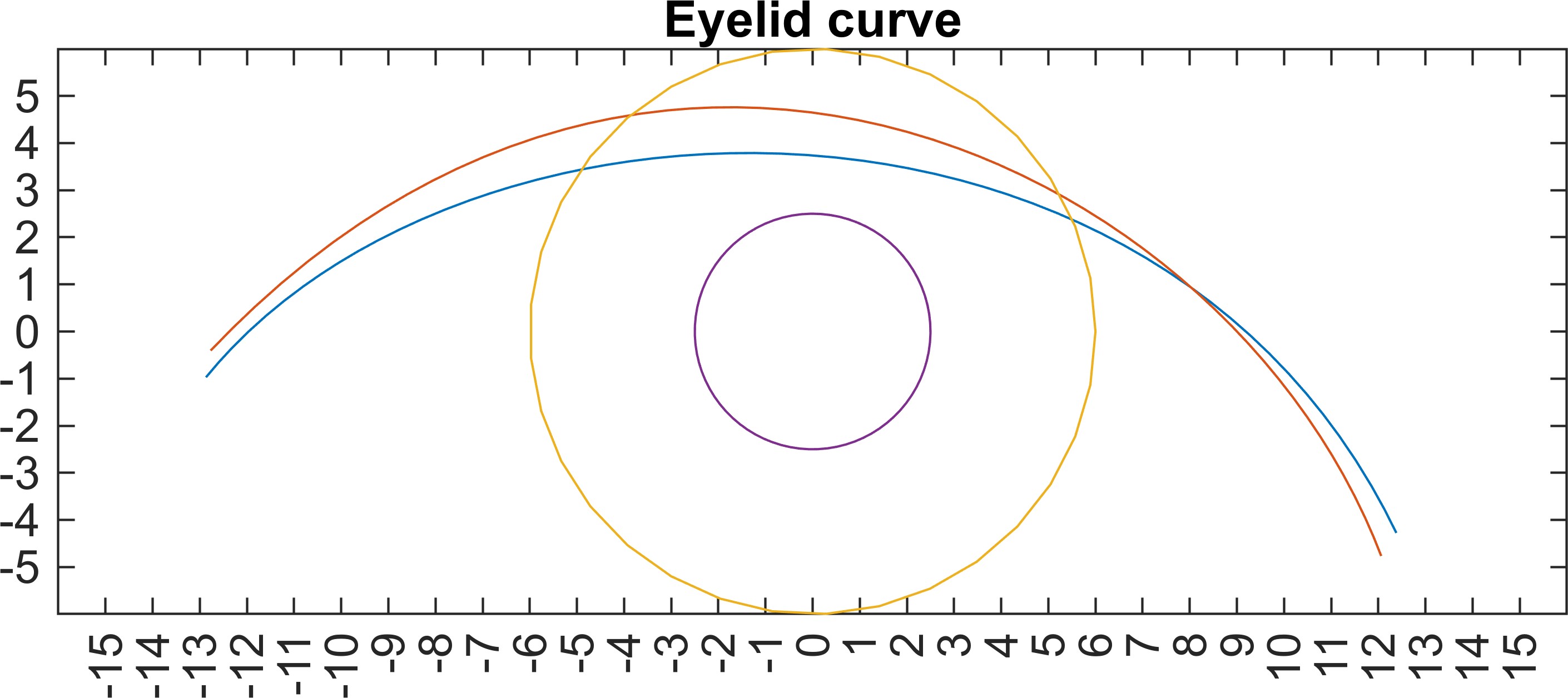

Supplement: S3 Appendix — (DOCX) [file pone.0316714.s003.docx]
